# Survey of Handlers of 158 Police Dogs in New Zealand: Functional Assessment and Canine Orthopedic Index

**DOI:** 10.3389/fvets.2019.00085

**Published:** 2019-04-16

**Authors:** Wendy I. Baltzer, Rebecca Owen, Janis Bridges

**Affiliations:** School of Veterinary Sciences, Massey University, Palmerston North, New Zealand

**Keywords:** police working dog, functional assessment, canine orthopedic index, musculoskeletal disease, gait

## Abstract

**Objectives:** To determine the functional assessment (FA) of fitness and Canine Orthopedic Index (COI) scores of 158 police dogs. The hypothesis was the dogs would have excellent fitness and no evidence of orthopedic disease regardless of age as reported by the handlers.

**Study Design:**Observational, prospective study.

**Sample Population:** Handlers of dogs >1 year of age in active duty or breeding/active duty.

**Methods:** COI and FA questionnaires were completed via e-mail. Fisher's Exact test for count data assessed scores by age group (<2 years, 2–5 years, >5 years); Wilcoxon Signed-rank test correlated COI parameters (stiffness, function, gait, quality of life) to FA.

**Results:** The dogs were 3.2 ± 2.4 (mean ± standard deviation) years-old, 96% were German Shepherds and 111 were male. 32% of dogs could hold the “Hup” position for no longer than 4 s and 8% frequently had difficulty with this task. Difficulty jumping into vehicles occurred in 1/3 of the dogs. Overall FA was impaired in 20% (score >8), abnormal in 15% (score = 5–7), and reduced (score = 1–4) in 36% of dogs. Only 29% had normal function (FA score = 0) and these were significantly younger (2.8 ± 1.7 years, *p* < 0.05) than impaired dogs (6.6 ± 2.2 years). COI stiffness score was abnormal in 37% (3.3 ± 2.2) and gait was abnormal in 41% (5.4 ± 4.0). Quality of life (QOL) was excellent in 69% of dogs. Stiffness for the <2 year-old group was 0.2 ± 0.8, for the 2–5 year-old group was 1.1 ± 2.0 and for the >5 year-old group was 3.2 ± 2.4. Gait score for the <2 year group was 0.8 ± 2.2, and for the 2–5 year group was 1.9 ± 3.2 and for the >5 year group was 6.0 ± 4.3. Quality of life was close to excellent for the <2 year-olds (0.3 ± 1.1) and 2–5 year-olds (0.8 ± 2.0) but the >5 year-olds scored higher (3.0 ± 2.5). Only the COI gait score correlated with the FA score (*p* = 0.30).

**Conclusions and Clinical Relevance:** Police dogs were reported by handlers to have good to excellent QOL, however, increasing age was associated with declining FA and COI scores.

## Introduction

Police dogs are trained to perform a variety of tasks including drug detection, suspect apprehension, tracking, protection, and search and rescue. These tasks require strength, balance, and coordination; therefore, the dogs must be physically fit to perform these duties and to prevent injury. Research involving injuries in these dogs as well as military dogs has found they are at increased risk of orthopedic disease compared to dogs living as pets (1–3) Police and military dogs serve similar functions and perform many of the same tasks. The New Zealand police dogs frequently scale 20–30 privacy fences in a single shift (Inspector Todd Southall, National Coordinator, New Zealand Police) and like military working and United States police dogs, develop acute and chronic musculoskeletal injuries (2, 4). Lameness, degenerative joint disease, and other musculoskeletal disorders are common in police dogs with a reported cumulative incidence of 36–44%, however, the effect of such disorders on performance has not been previously reported (3, 4). In addition, lameness is associated with age in police dogs (>5 years) and a common reason for retirement in up to 69% (4–6). New Zealand police dogs are retired at a median of 6.6 years of age and only 40% reach the planned or “goal” age of retirement of 8 years, most commonly due to orthopedic disease (6). There are currently no reports of the age of onset of the musculoskeletal disorders for which these working dogs are retired, either in the military or police, however, these disorders have an increased incidence in police dogs >5 years of age (4).

Physical conditioning programs have been developed in sporting dogs to improve performance and reduce injury, however these programs have had limited study of their effects. Within the last 15 years substantial research has been performed in humans indicating that training, and chronic loading reduces injury (7, 8). In addition, assessment of the subject's abilities and physical fitness can be used to predict which individuals are likely to become injured (9). Assessment of physical fitness in dogs, especially working dogs, has only recently begun. Preliminary findings in military dogs with degenerative lumbosacral disease found improved function and task performance following participation in a therapeutic exercise program (10). The authors developed a functional assessment completed by the handlers in a small group of dogs and identified a change in functional abilities following rehabilitation (10). This assessment was developed to determine the military dogs' ability to perform tasks such as the “Hup” maneuver (standing on hind limbs with forelimbs resting on a vertical surface), jumping into or out of a vehicle, climbing stairs or an A-frame, and sitting and standing repeatedly.

Few methods to assess dogs orthopedically have been validated, however, one such measure is the canine orthopedic index (COI) (11–13). This assessment tool was developed to reliably measure the owner's perception of outcome in dogs with orthopedic disease and has previously been validated in a placebo-controlled, blinded clinical trial of dogs with joint disease (11–13).

The objective of this study was to determine the functional fitness and orthopedic index of the New Zealand Police Dog Force and correlate these questionnaires to the age of the dogs. Our hypothesis was that these dogs would have excellent fitness and no evidence of orthopedic disease or functional impairment as determined by questionnaire of the dogs' handlers regardless of age.

## Materials and Methods

Police dog handlers of the New Zealand Police were contacted via email. Handlers with dogs >1 year of age in active duty or a combination of breeding/active duty were asked to complete the Canine Orthopedic Index (12, 13) (COI) and functional assessment (FA) questionnaires (Data Sheet 1 in Supplementary Material) (10). The name of the handler, dog name, age closest to the year, sex, desexing status, region of service, and breed were recorded.

COI scores were determined for each of the 4 parameters in the questionnaire: stiffness (4 questions), function (5 questions), gait (4 questions), and quality of life (3 questions) (12). The scores for each parameter were the sum of each question for the parameter. A score was given for each answer according to the following: “none,” “no problems,” “never,” or “excellent” received a score = 0. “Mild,” “mild problems,” “rarely,” or “very good” answers received a score = 1. “Moderate,” “moderate problems,” “occasionally,” or “good” answers scored a 2 and “severe,” “severe problems,” “frequently,” and “fair” scored a 3. Recorded answers of “extreme,” “extreme problems,” “constantly,” and “poor” were assigned a score of 4. The range of scores for stiffness were 0–16, for function 0–20, for gait 0–16, and for quality of life 0–12.

**Figure 1:**
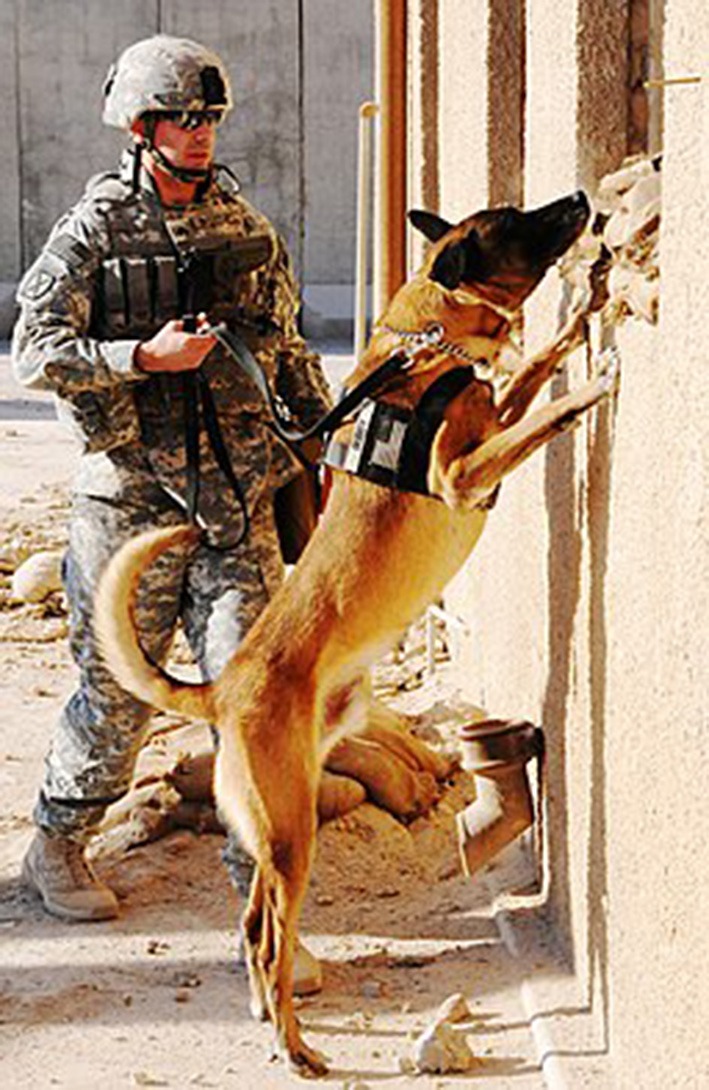
Military dog practicing the “hup” position or standing on its hind legs while forelimbs are resting on a door or vehicle for balance. The ability to perform this task was rated as “excellent” if the dog could remain in position for 8 or more seconds, “good” if holding position for 5–7 s, “fair” if able to hold position for 1–4 s and “poor” if unable to hold for 1 s or less or unable to perform the position (10). Photograph courtesy of The U.S. Army [Public domain], via Wikimedia Commons.

The overall FA score for each dog was the sum of the scores from all the questions. Question 1 was given the following value according to the answer chosen (ability to maintain the “Hup” position, Figure 1: “0 s” score = 4, “1–2 s” score = 3, “3–4 s” score = 2, “5–6 s” score = 1, “7 or more seconds” score = 0. Questions 2–10 were given the following values according to the answer chosen: “Never” score = 0, “Rarely” score = 1, “Sometimes” score = 2, “Frequently” score = 3, “Always” score = 4. A total score equal to 0 was classified as no evidence of impairment, whereas a total FA score equal to 1–4 was classified as potentially reduced fitness in the dog and the dog was classified as having evidence of borderline impairment if the total score was between 5 and 7. Dogs with a score >8 were classified as functionally impaired.

## Statistical Analysis

Correlation of the COI parameters to the FA score was analyzed by dividing the scores for each the dogs by the highest score possible. The scores were then tested for normality using the Shapiro-Wilk normality test. Because the data was not distributed normally the Wilcoxon Signed-rank test with continuity correction (non-parametric) was used to compare each of the 4 variables of the COI (Stiffness, Function, Gait, and Quality of Life scores) to the FA score.

The ages of the dogs was also not distributed normally. The dogs were divided into 3 groups for score comparison with 47 dogs in the ≤2 years of age group, 45 dogs in the 2–5 years, and 41 dogs in the >5 years of age group. Fisher's Exact test for count data was used to compare each of the COI scores and FA score by age group. Descriptive statistics were performed using spreadsheet software (Microsoft Office Excel 2010, Microsoft, Seattle, WA). Data were analyzed with the statistical software package R (R v 3.4.1, 2017 Foundation for Statistical Computing, Vienna, Austria). Statistical significance was set with a *p* < 0.05.

## Results

Questionnaires were completed by New Zealand police dog handlers for 158 police dogs. Not all handlers answered every question or completed both the COI and FA. Most handlers had never run their dog through a tunnel and did not answer this question on the FA questionnaire, therefore this question was removed from the analysis and the FA score for each dog was a total of the scores from the other 9 remaining questions.

The average age of the dogs was 3.2 ± 2.4 (mean ± standard deviation) years (4, 1–10 median, range) and 96% were German Shepherds (5 were Labradors and 2 were mixed-breeds). All German Shepherds were bred from a specific kennel owned and administered by the police with all dogs undergoing PennHip® assessment and elbow radiographs at 2 years of age. These assessments were not available for evaluation or inclusion in the analysis. There were 111 male intact dogs and 12 neutered males. Thirteen dogs were female, 16 were female-spayed, and in 6 dogs, sex was not specified.

COI stiffness score was abnormal in 37% with a score of 3.3 ± 2.2 [range 0 (normal)-16], function score was abnormal in 22% with 2.9 ± 2.1(range 1–16) mean score, and gait score was abnormal in 41% of dogs with a score of 5.4 ± 4.0 (range 1–20). Quality of life was excellent in 108 (69%) dogs who were significantly younger (3.2 ± 1.8) than the others (6.2 ± 2.2) with a mean QOL score of 3.5 ± 2.5 (range 1–12).

The handlers reported 70% of dogs have no difficulty climbing stairs or A-frame obstacles but 27% sometimes have difficulty and 3% frequently cannot perform these tasks. Sixty percent of the dogs can easily perform the “hup” position (standing on back legs for >8 s, Figure 1), but 32% can only hold the position for < 4 s and 8% frequently/always have difficulty. Thirty-two percent of the dogs sometimes had difficulty jumping into vehicles and 7% always needed assistance. When performing sit or down commands, 20% sometimes had difficulty while another 4% consistently did.

Overall function (FA score) was significantly impaired in 30 (20%, score > 8) dogs with abnormal function present in another 23 (15%, score = 5–7). Another 54 (36%, score = 1–4) dogs had potentially inadequate fitness. Only 44 (29%) dogs had normal function without any signs of impairment (Overall score = 0). Normally functioning dogs were significantly younger (2.8 ± 1.7 years) than borderline impaired or impaired dogs (6.6 ± 2.2 years), however dogs with reduced fitness were similar in age to normally functioning dogs.

There was a strong association between age group and the questionnaire scores when dogs were assigned to one of three groups (<2 years, 2–5 years, >5 years), Table 1. The cumulative FA score for the <2 year-old group was 1.8 ± 2.4 (mean ± standard deviation) and 3.2 ± 4.5 for the 2–5 year-old dogs; with the dogs >5 years of age scoring 8.3 ± 6.3 (*p* < 0.001). Similarily, scores for the COI parameters were lower in the <2 year-old group than the other groups (*p* < 0.001). Total stiffness score (not divided by the highest possible score) for the <2 year group was 0.2 ± 0.8, for the 2–5 year group was 1.1 ± 2.0 and for the >5 year group was 3.2 ± 2.4. COI function score for the <2 year group was 0.1 ± 0.7, and for the 2–5 year group was 0.2 ± 1.2 and for the >5 year group was 2.0 ± 2.1. Gait score for the < 2 year group was 0.8 ± 2.2, and for the 2–5 year group was 1.9 ± 3.2 and for the >5 year group was 6.0 ± 4.3. Quality of life was close to excellent for the <2 year-olds (0.3 ± 1.1) and 2–5 year-olds (0.8 ± 2.0) but was very good for the >5 year-olds (3.0 ± 2.5).

**Table 1:** Result of statistical analysis of each parameter of the COI (stiffness, function, gait, quality of life) compared to the FA (functional assessment questionnaire).

**Wilcoxon signed-rank test**	**V**	***p*-value**	**Result**
Stiffness and Functional assessment	1334.5	< 0.001	Reject H_0_
Function and Functional assessment	265	< 0.001	Reject H_0_
Gait and Functional assessment	2225.5	0.3038	Accept H_0_
QOL and Functional assessment	1376	0.0012	Reject H_0_

*The null hypothesis (H_0_) was that there was no difference in the values between parameters (summed and divided by the highest possible score) of the COI and functional assessment questionnaire as completed by handlers of active New Zealand police dogs. The null hypothesis was rejected for all the COI parameters except the gait assessment which was similar to the functional assessment results. V, the sum of the signed rank statistic*.

**Figure 2:**
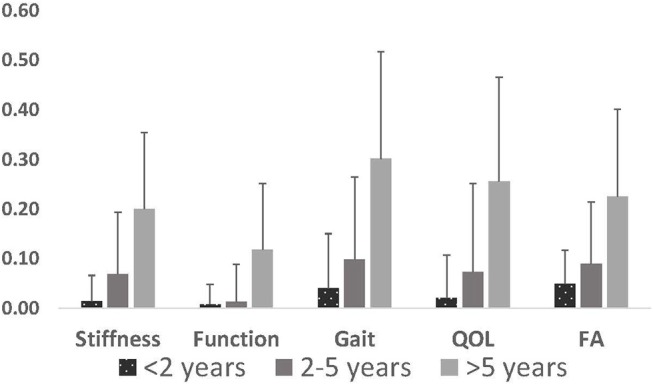
Mean ± standard deviation of scores for each of the questionnaire parameters for the COI (stiffness score, function score, gait score, quality of life) and functional assessment. Y axis values are the sum of the questions for each score divided by the highest possible score for each parameter. Age significantly affected score for all parameters reported (*p* < 0.001). QOL, quality of life; FA, functional assessment.

The COI scores for stiffness, function, and quality of life did not correlate with the cumulative FA score (*p* < 0.001), however the FA score did correlate with the gait score of the COI questionnaire (*p* = 0.30) and may provide similar information, at least for some parameters in working dogs as the COI (Figure 2).

## Discussion

In New Zealand, police dogs have an overall excellent quality of life and most were able to complete the functional tasks in the questionnaires completed by their handlers. Approximately 20–25% of the police dogs had indications, as reported by their handlers, that function, fitness, and specific task abilities were impaired with 3–4% consistently displaying difficulty with tasks such as climbing stairs, repeatedly sitting and standing and jumping into and out of vehicles. Surprisingly, 32% of the dogs could hold the “hup” position for only 4 s or less. Weakness in hind limb musculature as a result of spinal disease such as lumbosacral disease, gracilis/semitendinosis myopathy or osteoarthritis could have accounted for the weakness reported in these dogs as these problems have been previously reported in German Shepherds working in the military and with the police (3, 6, 14–16). Orthopedic disease is common in dogs in the general population as well as in working police dogs and the COI has been validated as a questionnaire for owners to assess orthopedic disease in pet dogs (13, 17). The abnormal COI findings in the dogs in this report may indicate that the COI could be a useful tool, along with physical examination and diagnostics, in the determination of physical fitness in working dogs and warrants further investigation. While none of the dogs in this study were currently under veterinary care for orthopedic disease, many may have had musculoskeletal injuries or other orthopedic disorders. This preliminary study did not determine the presence or severity of orthopedic disease present in the dogs with elevated scores in the COI and FA questionnaires, however future studies utilizing these tools may provide further understanding of the relationship between physical fitness in working dogs and their ability to perform assigned tasks as well as injury risk.

Because the COI has been validated and extensively studied, correlation of at least one parameter with the unvalidated FA questionnaire (gait score) indicates that the FA may have some utility for assessment of musculoskeletal disorders in dogs with an increased incidence of musculoskeletal disease and injury such as police and military dogs. The gait component of the COI questionnaire assesses the frequency of limping observed by the owner and whether clinical signs of gait abnormalities are present the day following increased exertion by the dog (11). Police dogs often participate in increased activity that may result in fatigue and even injury that is evidenced the day following the activity and therefore may be a more sensitive component of the COI for working dogs. The FA also attempts to determine the incidence of fatigue and gait abnormalities following exertion and specific tasks performed by police and military dogs, therefore correlation of these two scores may not be surprising (10). Further validation and investigation of its reliability as well as the ability of the FA to detect changes in function in working dogs may be warranted.

While neither the COI nor the FA were developed specifically to determine physical fitness or fitness of the police dog to perform its official tasks, the results presented here indicate that these highly trained dogs may have signs of intermittent over-exertion or even injury and that as these dogs age, the likelihood of reduced function and signs of orthopedic disease increase. As a police dog's age increases (>2 years), physical fitness may be reduced and function may become impaired with an increase in the likelihood of injury in dogs >5 years of age (4). Sixty nine percent of New Zealand police dogs are retired due to degenerative musculoskeletal disease resulting in an inability to perform assigned tasks at a mean age of 7 years (6). The results of the current study agree with the previous study that as these dogs age, their functional abilities decline and their incidence of orthopedic disease-related signs increases. The results of the current study go further and indicate that for many of the police dogs, the reduced ability to perform assigned tasks, development of gait abnormalities and prolonged fatigue following exertion begins as early as 2–5 years of age and that by >5 years of age significant impairment occurring more than just occasionally has developed. Fortunately, dogs with mildly increased COI and FA were young and of similar age as dogs with excellent function (*p* > 0.05) scores which may be explained by potentially impaired fitness. Because these dogs were <5 years of age and less likely to have permanent musculoskeletal disorders (4), there may be room to improve their fitness even as young dogs.

## Study Limitations

There were limitations identified in this study including the reliance of the handlers to accurately assess the dogs and their functional abilities. The handlers were instructed to answer the questions honestly and were told that the administration would not be informed of the results for individual dogs in order to reduce bias, however, this may still have affected the results. Not all handlers completed the entire questionnaire nor did all handlers report the sex or age of the dogs and this decreased the number of dogs in the statistical analysis. A larger number of dogs of differing ages would ideally be investigated with general, orthopedic, and neurological physical exams obtained by veterinarians at the time of handler completion of the questionnaires in order to further interpret the findings of the present study. In addition, the functional assessment questionnaire has not been validated, therefore results must be interpreted with caution. Ideally the results from the handlers would have been compared to concurrent assessment with orthopedic, neurologic and diagnostic imaging exams and evaluation of lameness in these dogs. Future studies are warranted to validate these questionnaires and the handlers' abilities to assess working dogs specific task function as well as physical fitness.

## Conclusions and Clinical Relevance:

The findings of this study support the relationship of increasing age on declining performance of assigned tasks in working dogs. Only 29% of police dogs have functional scores without deficits reported by their handlers. Forty-one percent of these dogs had gait abnormalities and COI stiffness score was abnormal in 37% of dogs. This is the first report, to the authors' knowledge, that police dogs as young as 3 years of age may have intermittent to occasional signs of reduced function and increased canine orthopedic index scores. Investigation into the nature of the elevated scores and reduced function may provide methods for prevention of injury in these dogs. Development of further methods to determine physical fitness, risk of injury, and early impaired function in working dogs may prolong their working life, improve performance and reduce serious injuries.

## Data Availability

All datasets generated for this study are included in the manuscript and/or the supplementary files.

## Ethics Statement

This study was carried out in accordance with the recommendations of the Massey University School of Veterinary Sciences policies on animal care and use. No prior Animal Institutional Care Committee approval was obtained because no animals were used in this research.

## Author Contributions

WB and RO participated in questionnaire acquisition and data collation. WB wrote the manuscript and JB performed statistical analysis.

### Conflict of Interest Statement

The authors declare that the research was conducted in the absence of any commercial or financial relationships that could be construed as a potential conflict of interest.

## Abbreviations

FA: functional assessment of fitness
COI: canine orthopedic index
QOL: quality of life
SD: standard deviation.
