# Supplementary material for: Survey of Handlers of 158 Police Dogs in New Zealand: Functional Assessment and Canine Orthopedic Index
Source: Front Vet Sci. 2019 Apr 16;6:85. doi: 10.3389/fvets.2019.00085 (PMC6478039; doi:10.3389/fvets.2019.00085)
Supplement: Supplementary file 1 [file Data_Sheet_1.PDF]

Handler: First Name______________ Last Name_____________________
Region of Service
Dog: Name_________________ Sex (Circle One): Male Female
Spayed/Neutered? (Circle One): Yes No
Age (round to the nearest year)_______
Breed: ____________________________

Functional Assessment
The following questions are about your evaluation of your police dog's performance during a training session. Please read the following questions and answer each one to the best of your ability. Provide only one answer for each question.

1. How long does the dog stay in the "hup" position ("hup" =**standing up on back legs**) during the evaluation routine?
a. 0 seconds, the dog refuses to "hup" when I ask
b. 1-2 seconds
c. 3-4 seconds
d. 5-6 seconds
e. 7 or more seconds

2. Does the dog show any difficulty "jumping" or "hupping/standing up" each time you ask, to a distance of at least three feet off the ground at the dog's shoulder level? Difficulty may be defined as hesitation in going into the hup, repeatedly unsuccessful attempts at rising into the **"hup," refusal to "hup" or crying out when performing the activity.**
a. Never
b. Rarely
c. Sometimes
d. Frequently
e. Always

3. Does the dog show any difficulty in jumping INTO a vehicle? Difficulty may be defined as hesitation, inability to complete the jump the first time, crying out when performing the activity, or slipping or falling during or after the jump.
a. Never
b. Rarely, Hesitates
c. Sometimes, inability to completely jump into vehicle
d. Frequently, slips or falls
e. Always

4. Does the dog show any difficulty in jumping OUT OF a vehicle? Difficulty may be defined as hesitation, crying out when performing the activity, or slipping or falling during or after the jump.
a. Never
b. Rarely
c. Sometimes
d. Frequently
e. Always

5. Does the dog show any difficulty in climbing the staircase on an obstacle course? Difficulty may be defined as hesitation, repeatedly attempting to get down, refusal to climb, crying out when performing the activity, or slipping or falling during the climb.
a. Never
b. Rarely
c. Sometimes
d. Frequently
e. Always

6. Does the dog show any difficulty in climbing up or down the A-frame on an obstacle course? Difficulty may be defined as hesitation, repeatedly attempting to get down, refusal to climb, crying out when performing the activity, or slipping or falling during the climb.
a. Never
b. Rarely
c. Sometimes
d. Frequently
e. Always

7. Does the dog show any difficulty performing 1-meter jumps on an obstacle course? Difficulty may be defined as hesitation, refusal to jump, crying out when performing the activity, not clearing the jump, or slipping or falling upon landing.

a. Never
b. Rarely
c. Sometimes
d. Frequently
e. Always

8. Does the dog show any difficulty sitting from a standing position and/or standing from a sitting position when performing this exercise 5 times in a row rapidly? Difficulty may be defined as hesitation or reluctance to down/stand, refusal to down or stand, or delayed or awkward changes in position when going into a down or rising into a stand.
a. Never
b. Rarely
c. Sometimes
d. Frequently
e. Always

9. Does the dog show any difficulty rising from a down position and/or going into down from a standing position when performing this exercise 5 times in a row rapidly? Difficulty may be defined as hesitation or reluctance to sit/stand, refusal to sit or stand, or delayed or awkward changes in position when sitting or rising into a stand.
a. Never
b. Rarely
c. Sometimes
d. Frequently
e. Always

10. Does the dog show any difficulty performing a 2-meter tunnel crawl on an obstacle course? Difficulty may be defined as hesitation in going into or coming out of the tunnel, repeatedly unsuccessful attempts at getting down or rising, refusal to enter the tunnel or get up from the crawl position, or crying out when performing the activity.
a. Never
b. Rarely
c. Sometimes
d. Frequently
e. Always

Today's Date: ☐☐ / ☐☐ / ☐☐
Month / Day / Year

(Study ID) ☐☐☐☐

Canine Orthopedic Index

Description of Stiffness

The following questions concern the amount of joint stiffness your dog has experienced in the **past 7 days**. Stiffness is the restriction or slowness in the ease with which your dog moves his/her joints.
Please select **one** answer for each question below.

1. How severe is your dog's stiffness after first wakening **in the morning**?

○ None ○ Mild ○ Moderate ○ Severe ○ Extreme

2. **Later in the day**, how severe is your dog's stiffness after lying down for at least 15 minutes?

○ None ○ Mild ○ Moderate ○ Severe ○ Extreme

3. How much of a problem does your dog have **rising to standing** after lying down for at least 15 minutes?

○ No problems ○ Mild problems ○ Moderate problems ○ Severe problems ○ Extreme problems

4. In general, over the past 7 days, how much difficulty has your dog had with his or her joints?

○ None ○ Mild ○ Moderate ○ Severe ○ Extreme

Description of Function

Please indicate how much of a problem each of the following activities has been for your dog over the **past 7 days.**
Please select **one** answer for each question below.

5. **Jumping up** (as in getting into the car or onto the bed) ?

○ No problems ○ Mild problems ○ Moderate problems ○ Severe problems ○ Extreme problems

6. **Jumping down** (as in getting out of the car or off of the bed) ?

○ No problems ○ Mild problems ○ Moderate problems ○ Severe problems ○ Extreme problems

7. **Climbing up** (as in stairs, ramps or curbs) ?

○ No problems ○ Mild problems ○ Moderate problems ○ Severe problems ○ Extreme problems

8. **Climbing down** (as in stairs, ramps or curbs) ?

○ No problems ○ Mild problems ○ Moderate problems ○ Severe problems ○ Extreme problems

Today's Date: ☐☐ / ☐☐ / ☐☐
Month Day Year

(Study ID) ☐☐☐☐

Canine Orthopedic Index

Description of Gait

The following questions concern your dog's gait over the **past 7 days**.
Gait refers to the manner in which your dog uses its legs as it moves.
Please select **one** answer for each question below.

9. On average, how severe was your dog's limp **during** mild activities (such as short walks)?

○ None ○ Mild ○ Moderate ○ Severe ○ Extreme

10. On average, how severe was your dog's limp **during** moderate activities (such as long walks, playing or running)?

○ None ○ Mild ○ Moderate ○ Severe ○ Extreme

11. How often did your dog limp **the day after** moderate activities (such as long walks, playing or running)?

○ Never ○ Rarely ○ Occasionally ○ Frequently ○ Constantly

12. How often have you been aware of your dog's joint problems?

○ Never ○ Rarely ○ Occasionally ○ Frequently ○ Constantly

13. How often did your dog 'pay' for over-activity, with increased pain or stiffness the following day?

○ Never ○ Rarely ○ Occasionally ○ Frequently ○ Constantly

Description of Quality of Life

Please select **one** answer for each question below.

14. In the past 7 days, what has been your level of concern that your dog's joint problems will shorten his or her life?

○ None ○ Mild ○ Moderate ○ Severe ○ Extreme

15. In the past 7 days, what has been your level of concern that your dog is generally slowing down?

○ None ○ Mild ○ Moderate ○ Severe ○ Extreme

16. Overall, how would you rate your dog's quality of life over the past 7 days?

○ Excellent ○ Very Good ○ Good ○ Fair ○ Poor
